# Authors' Responses to Peer Reviews of “Patterns of Physical Activity Among University Students and Their Perceptions About the Curricular Content Concerned With Health: Cross-sectional Study”

**DOI:** 10.2196/37206

**Published:** 2022-04-29

**Authors:** Arun Kumar Verma, Girish Singh, Kishor Patwardhan

**Affiliations:** 1 Department of Kriya Sharir Faculty of Ayurveda, Institute of Medical Sciences Banaras Hindu University Varanasi India; 2 Centre of Biostatistics Institute of Medical Sciences Banaras Hindu University Varanasi India

**Keywords:** physical activity, university students, university, exercise, students, inactive, curricula, healthy lifestyle, higher education

*This is the authors’ response to peer-review reports for “Patterns of Physical Activity Among University Students and Their Perceptions About the Curricular Content Concerned With Health: Cross-sectional Study.”*

## Round 1 Review

### Reviewer E [1]

#### General Comments

It is very positive to see analysis of physical activity in different populations and different age groups, and this paper [2] is a very welcome study in terms of physical activity in India and in relation to students. This is an important area as, when trying to engender habits and physical activity across the life span, it is in the younger age groups where sustained impact can be made. However, I feel that this paper addresses the issue quite superficially and would benefit from more in-depth analysis.

Reply: Thanks for these encouraging remarks. We have tried our level best to address the issues you have pointed out.

#### Specific Comments

##### Major Comments

1. Throughout the paper, there is no point at which the categories of inactive, active, and highly active are defined—this is a major omission as it is impossible to gauge how this compares to, for example, World Health Organization or other national guidelines in terms of minutes physical activity per week or metabolic equivalent minutes (apologies if this is indeed in the paper and I have missed it).

Reply: Thanks for pointing this out. We have now added a section that describes the categorical classification.

2. Demographics: although the authors should be commended for looking at differences between gender and age, there is no comment on socioeconomic status. For example, earlier in the paper, when describing the university, it would be useful to know what the demographics of the student population are (ie, do they represent general society or higher socioeconomic status?) This is important, as socioeconomic status (in the United Kingdom at least) is a major driver of physical activity. It would be useful for the reader as to how the subject population compares with the general population.

Reply: This has now been included, both in introduction and discussion sections. Word Health Organization suggests that economically developed regions are likely to show trends of physical inactivity. This seems to hold true in our case as the region is economically not a very developed one.

3. It is unclear to me how the metabolic equivalent minutes values of the subject population relate to that of the general population, and internationally. Over 4000 metabolic equivalent minutes per week is several times over World Health Organization guidance, and I would expect some analysis of how and why this might be the case.

Reply: A paragraph in discussion section has now been included that lists the limitations of the study and also points out other aspects that one needs to consider while interpreting our results.

4. In the discussion, there is a lot of description of the results from previous studies, and comparison with the current study, but without any analysis as to why there are similarities or differences. I also felt there was no real incorporation of the perceptions into the discussion, and no real analytical depth.

Reply: This analysis has now been added.

5. In the discussion, there is no real discussion of the limitations of the approach used, and no contextual framing of the findings.

Reply: New paragraphs have been added to address this.

##### Minor Comments

Abstract; objectives: Line beginning “the study also aims...” not quite clear: perhaps “This study also aims to capture student perceptions about the balance between curricular activities and leading a physically active lifestyle...”?

Reply: This has been corrected.

Introduction: “being overweight” rather than “overweight.”

Reply: This has been corrected.

It would be useful to describe briefly what the few studies regarding students show.

Methods: validation of the new tool—more information on this would be useful: does the Cronbach alpha number represent test-retest reliability? In which case, how was validity measured?

Reply: Thanks for pointing this out. Yes, we tested the tool for its reliability and did not validate it. We have corrected this.

Data collection and data entry: “written consent was obtained from each of the participants.”

Reply: This has been corrected.

How were outliers excluded? How did the authors define “erratic entries”? Is this according to International Physical Activity Questionnaire cleaning criteria?

Reply: Yes, this was done as per the guidelines of data processing available for International Physical Activity Questionnaire—Long Form tool. This has been clarified.

Views and opinions of the students: I would want more description of the items where there was discrepancy.

Reply: We have tried our level best to do this. However, since the items are now included in each of the tables directly, this description has become self-explanatory.

Table 8: There is a comment at the end of this section regarding why the authors feel students in different faculties are performing different levels of physical activity. This belongs in the discussion.

Reply: This sentence has now been moved to discussion section.

Discussion: the study on pooled data: was this from university students?

Reply: No. These pooled data are from population-based studies. We included them to have a comparative perspective.

Tables: Table 4—why was a Mann-Whitney test used if the data presented are in mean (SD) (ie, if the data are nonparametric, shouldn’t the median IQR be used?).

Reply: When the data do not follow normal distribution, then Mann-Whitney test may be used for intergroup comparison based on rank. Median and IQR are summary measures, which may also be given; however, they are not a test of significance, and in such case, test for median (sign test) may be calculated. We prefer giving the results based on Mann-Whitney test because significance can be inferred.

Table 6-8: it would be helpful to have the questions in the table to enable the reader to better see how they relate.

Reply: This has been done now. Each item has now been included in the table.

### Reviewer F [3]

#### General Comments

This paper [2] was about the physical activity pattern of university students aiming at measuring this for the first time systematically as well as creating a new tool in order to have more accurate results. The authors collected a large body of data over several years, which gives an accurate and realistic perspective of the physical activity patterns of university student in India. It was an honour to read this remarkable job the authors did over the years.

Reply: Thanks for these encouraging remarks!

#### Specific Comments

##### Numbered Comments

1. I find the Introduction part quite short compared to the literature mentioned in Discussion. I learned more about the literature from Discussion than from the Introduction. I’d suggest writing a slightly longer introduction with details on activity patterns of different age groups. This could also point to the missing age group data this paper focuses on.

Reply: We appreciate your observation. We have now elaborated the introduction part.

2. The authors mention in the first paragraph of the introduction, “an increased engagement with video games, cell phones, television, computers, and social media are possibly some of the important contributing factors to this trend among youth.” I’d write in more detail about this or have a bigger emphasis on this perspective in the paper as the manuscript was submitted to the Journal of Medical Internet Research both in the introduction and in the discussion.

Reply: Thanks for this suggestion. We have now elaborated on these aspects both in the introduction section and in discussion.

3. The authors mention in the methods, in the study design and sampling, “time and other limitations”. I’d rather mention these in the limitations part of the discussion, and I’d explicitly say what are the other limitations not listed here. The authors write “approximately 4600 students” in this section. On the other hand, I read the exact number later. I’d suggest writing the exact number because it is accessible.

Reply: These issues have now been addressed accordingly.

4. In the “translation and revalidation” part, the authors mention a “professional” who did the translation and retranslation. I find it important what professional they were? Translators, interpreters, psychologists, English teachers? What profession did they have? You also mention “suitable corrections were made.” What does this mean? Were certain items deleted based on certain criteria? I am not sure I understand the last sentence, “both the versions of the tool were used in the study to collect data based on student preference.” I wonder if it would be possible to make it clear what two version were used?

Reply: Clarification on this aspect has now been provided in the manuscript.

5. In the “development of a new tool,” I was wondering in what language did you state these questions? My understanding is that in Hindi. I’d suggest writing it explicitly. I also wonder why these 5 items were used? what was the process of creating these items? Were there possibly more, and then you deleted the ones that did not work? What did you base your decision on to use these exact 5 items?

Reply: The clarification has been now provided in the manuscript.

6. In the “validation of the new tool,” you write “acceptable range.” I suggest giving a literature reference on what you based your decision on, what is acceptable, and what is not. I read the manuscript, and you reported the Cronbach alpha. In my understanding, this means the tool is reliable; however, was not validated. For example, correlation with other tools.

Reply: Appropriate reference has now been added. As you have rightly pointed out, the tool was tested for reliability and was not validated. We have rectified this error.

7. The authors reported the data collection was between 2016 and 2019. This is a long stretch of time, and physical activity patterns can change in different groups year by year. I’d suggest for the authors to consider a statistical analysis on the data year by year. For example, people who filled out the questionnaire in 2016, the ones in 2017, and so on.

Reply: We did not address this as it would not give any additional inputs. However, a clarification to this effect has been included in the discussion section.

8. I read in the results you reported significant and not significant results. I’d consider writing a sentence about the direction of significant results. For example, “the difference between physical activity of students of different age groups was statistically significant.” I’d find useful to read a sentence about which age group was more active and which one less active.

Reply: This has now been addressed suitably.

9. I’d find it useful if I could read the results in hour as well, beside reading it in minutes. As far as I understand, the tool used reports in minutes. However, it would be easier to read if I could read it also in hours.

Reply: We did not do this as International Physical Activity Questionnaire—Long Form questionnaire captures the activities in terms of minutes only. Conversion into hours is not usually recommended. Neither in the literature we find such reporting.

10.I’d suggest using the last sentence of the results in the discussion. “Hence, it ca be presumed that the students in these faculties get some or the other kind of motivation to lead a physically active lifestyle as a part of their curriculum.”

Reply: Thanks! We have done this.

11. The authors write in the discussion, “this is possibly one of the first studies from India that looks at psychical activity…”. I’d suggest not using the phrase “possibly.” After having read the literature on India about psychical activity of students, it can be said if this is the first or one of the first papers reporting on the matter.

Reply: We have corrected this.

12. I’d find it useful to have a section for abbreviations.

Reply: This has been done now.
